# Analysis of Cardiorespiratory Fitness in Early Adulthood and Midlife With All-Cause Mortality and Fatal or Nonfatal Cardiovascular Disease

**DOI:** 10.1001/jamanetworkopen.2023.0842

**Published:** 2023-02-28

**Authors:** Kelley Pettee Gabriel, Byron C. Jaeger, Cora E. Lewis, Stephen Sidney, Erin E. Dooley, Mercedes R. Carnethon, David R. Jacobs, Bjoern Hornikel, Jared P. Reis, Pamela J. Schreiner, James M. Shikany, Kara M. Whitaker, Alexander Arynchyn, Barbara Sternfeld

**Affiliations:** 1 Department of Epidemiology, The University of Alabama at Birmingham, Birmingham; 2 Wake Forest School of Medicine, Department of Biostatistics and Data Science, Wake Forest University, Winston-Salem, North Carolina; 3 Division of Research, Kaiser Permanente Northern California, Oakland; 4 Feinberg School of Medicine, Department of Preventive Medicine, Northwestern University, Chicago, Illinois; 5 Division of Epidemiology and Community Health, University of Minnesota, Minneapolis; 6 Division of Cardiovascular Sciences, National Heart, Lung, and Blood Institute, Bethesda, Maryland; 7 Division of Preventive Medicine, The University of Alabama at Birmingham, Birmingham; 8 Department of Health and Human Physiology, University of Iowa, Iowa City

## Abstract

**Importance:**

Optimizing cardiovascular fitness is a prevention strategy against premature death and cardiovascular disease (CVD) events. Since this evidence has largely been established in older populations, the importance of cardiorespiratory fitness during earlier periods of adulthood remains unclear.

**Objective:**

To examine the association of early-adulthood cardiorespiratory fitness and percentage of early-adulthood cardiorespiratory fitness retained during midlife with subsequent risk of all-cause mortality and CVD-related morbidity and mortality overall as well as by sex and race.

**Design, Setting, and Participants:**

This retrospective population-based cohort study analyzed data from the Coronary Artery Risk Development in Young Adults (CARDIA) study, an ongoing prospective cohort study conducted at field center clinics in Birmingham, Alabama; Chicago, Illinois; Minneapolis, Minnesota; and Oakland, California. Participants in the CARDIA study were aged 18 to 30 years when they completed the baseline graded exercise test protocol in 1985 to 1986 and have since undergone follow-up examinations biannually and every 2 to 5 years. Data were collected through August 31, 2020, and were analyzed in October 2022.

**Exposures:**

Cardiorespiratory fitness was estimated from a symptom-limited, maximal graded exercise test protocol conducted at baseline and at year 7 and year 20 follow-up examinations.

**Main Outcomes and Measures:**

All-cause mortality and combined fatal and nonfatal CVD events were obtained since year 20 of follow-up examinations through August 31, 2020. Cox proportional hazards regression models were used to estimate hazard ratios (HRs) for each primary exposure with each outcome.

**Results:**

A total of 4808 participants (mean [SD] age at baseline, 24.8 [3.7] years; 2670 females [56%]; 2438 Black individuals [51%]) were included in the sample. During 68 751 person-years of follow-up, there were 302 deaths (6.3%) and 274 CVD events (5.7%) since year 20. Every 1-minute increment in cardiorespiratory fitness at baseline was associated with a lower risk of all-cause mortality in females (HR, 0.73; 95% CI, 0.64-0.82) and males (HR, 0.87; 95% CI, 0.80-0.96). Every 5% increment in cardiorespiratory fitness retained through year 20 was associated with a lower risk of all-cause mortality (HR, 0.89; 95% CI, 0.79-0.99), but no evidence of effect modification by race or sex was found. Every 1-minute increment in cardiorespiratory fitness at baseline was associated with a lower risk of fatal or nonfatal CVD (HR, 0.89; 95% CI, 0.82-0.96), and the estimated HR per 5% increment in cardiorespiratory fitness retained throughout midlife was 0.89 (95% CI, 0.78-1.00), with no evidence for interaction by race or sex.

**Conclusions and Relevance:**

This cohort study found that higher early-adulthood cardiorespiratory fitness and greater retention of early-adulthood cardiorespiratory fitness throughout midlife were associated with a lower risk of premature death and CVD events. Additional research is needed to clarify the association of cardiorespiratory fitness timing across the life course with risk of clinical outcomes.

## Key Points

**Question:**

Is cardiorespiratory fitness during early adulthood, and its maintenance through midlife, associated with subsequent clinical outcomes?

**Findings:**

In this cohort study of 4808 participants in the CARDIA study, every 1 minute of higher early-adulthood cardiorespiratory fitness was associated with a lower risk of premature death, and every 5% increase in cardiorespiratory fitness retained throughout midlife was associated with a lower risk of all-cause mortality. Early-adulthood cardiorespiratory fitness was also associated with a lower risk of nonfatal or fatal cardiovascular events.

**Meaning:**

Findings of this study support the importance of higher cardiorespiratory fitness during early adulthood and throughout midlife for improved outcomes and suggest the need for additional research to clarify the association of cardiorespiratory fitness timing with risk of clinical outcomes.

## Teaser

This cohort study evaluates whether cardiovascular fitness during the early periods of life is associated with morbidity and mortality outcomes and whether the outcomes vary by sex and race.

## Introduction

Cardiorespiratory fitness is an attribute that reflects coordination of multiple physiological systems, including cardiovascular, respiratory, and musculoskeletal.^1^ While several epidemiological studies have demonstrated the inverse association of cardiorespiratory fitness with risk of all-cause mortality and nonfatal or fatal cardiovascular disease (CVD),^2,3,4^ most studies have estimated these associations prospectively using a single-exposure estimate of cardiorespiratory fitness.^2,5,6^ Across studies, the timing of the cardiorespiratory fitness assessment spanning the adult life course has varied, with midlife being the most common period,^2^ likely because of its proximity to chronic disease outcomes and mortality. While this period has led to consistency in findings, it does not determine whether there are critical windows prior to midlife that are associated with risk of premature death and CVD events.

There are limited longitudinal data on changes in cardiorespiratory fitness. Prior findings from the Coronary Artery Risk Development in Young Adults (CARDIA) study suggested an accelerated decline in cardiorespiratory fitness^7^ that exceeded results reported in a prior longitudinal study.^8^ Specifically, estimated maximal cardiorespiratory fitness declined from 4.6% from age 20 to 25 years to approximately 10% every 5 years from age 30 to 50 years.^7^ It is unclear whether cardiorespiratory fitness during early adulthood and changes in cardiorespiratory fitness throughout midlife are associated with subsequent health risks.

This research gap was addressed by leveraging the CARDIA study, a well-characterized, longitudinal, prospective cohort study that conducted a symptom-limited graded exercise test (GXT) protocol at baseline (1985-1986, with participants aged 18-30 years) and follow-up examinations in year 7 (1992-1993) and year 20 (2005-2006). Ongoing cohort follow-up occurs every 6 months for updates of contact information and vital status as well as for annual ascertainment of hospitalizations, outpatient procedures, and other changes in health status. The primary objective of the present study was to examine the association of early-adulthood cardiorespiratory fitness at baseline and percentage of early-adulthood cardiorespiratory fitness retained in midlife at the year 20 follow-up with subsequent risk of all-cause mortality and CVD-related morbidity and mortality. This association was also examined across and within sex and race subgroups.

## Methods

### Study Design and Participants

The CARDIA study includes 5115 males and females, who self-identified their race as Black or White, aged 18 to 30 years who completed the baseline GXT in 1985 to 1986 at a field center clinic in Birmingham, Alabama; Chicago, Illinois; Minneapolis, Minnesota; or Oakland, California.^9^ In addition to biannual follow-up, participants completed an in-person follow-up examination every 2 to 5 years (years 2, 5, 7, 10, 15, 20, 25, 30, and 35). Standardized questionnaires and protocols were used for data collection and are publicly available on the CARDIA study website.^10^ The present cohort study received approval from the institutional review board at each field center clinic, and written informed consent was obtained from all participants. We followed the Strengthening the Reporting of Observational Studies in Epidemiology (STROBE) reporting guideline.

### Primary Exposure: Cardiorespiratory Fitness

Cardiorespiratory fitness was estimated based on exercise duration (in minutes) achieved during a maximal, symptom-limited protocol consisting of up to nine 2-minute stages of increasing workload, ranging from 4.1 to 19.0 MET (metabolic equivalent task).^11^ A GXT was considered to be valid if the participant achieved at least 85% of the age-predicted maximal heart rate using the CARDIA study formula.^12^ Heart rate, blood pressure, and electrocardiogram were collected at rest, during the last 30 seconds of each stage, at peak effort, and every minute during the recovery period. The participant’s rating of perceived exertion, using the Borg scale (range: 6-20, with higher scores indicating higher perceived exertion),^13^ was collected near the end of each stage and at peak effort. A calibration equation was used at the year 7 follow-up examination to correct exercise duration values from a single clinic that permitted use of treadmill handrails^12^ during exercise. Results from a prior study^7^ found comparable results when these tests were excluded. Participants with at least 1 valid GXT were included. When data were missing, a linear mixed model was used to estimate treadmill duration.^7^ These model-based estimates were adjusted for age and included participant-specific intercepts and slopes to account for individual heterogeneity in cardiorespiratory fitness over time. The GXT duration at baseline (ie, cardiorespiratory fitness in early adulthood) and the relative level (in percent) of baseline GXT duration retained at year 20 (ie, cardiorespiratory fitness in midlife) were used for analysis.

### Primary Outcomes: All-Cause Mortality and Fatal and Nonfatal CVD

As part of the cohort follow-up, death certificates and autopsy reports (if conducted) were requested for all deaths, and medical records were requested for all suspected CVD events. For participants who could not be contacted, vital searches were performed annually using a variety of resources, including obituary search engines, US Social Security Death Index, and/or next of kin or friends. The National Death Index searches are completed every 5 years. Two physician members of the CARDIA Endpoints Surveillance and Adjudication Subcommittee independently adjudicated each record for underlying cause of death or potential event,^14,15,16,17,18^ and major disagreements were resolved by the entire committee.^19^ Data on deaths from any cause and on nonfatal (myocardial infarction, cardiac revascularization, acute coronary syndrome, congestive heart failure, stroke, transient ischemic attack, carotid artery disease, or peripheral artery disease) or fatal (coronary heart disease, stroke, or atherosclerotic or nonatherosclerotic cardiac disease) CVD events after the year 20 follow-up examination were obtained through August 31, 2020. Approximately 90% of the surviving cohort has been directly contacted within the past 5 years.

### Covariates

Covariates were selected a priori based on a literature review and/or biological plausibility for confounding the associations of cardiorespiratory fitness with all-cause mortality and CVD-related morbidity and mortality. Sex assigned at birth (male or female), self-identified race (Black or White; per design, the CARDIA study recruited only individuals identifying as Black or White), and enrollment in a CARDIA study clinic (to account for geography) were included. Time-varying measures included educational level (≤high school diploma or General Educational Development [GED] certificate or ≥associate’s degree); difficulty paying for basics such as food, medical care, and heating (somewhat hard, hard, very hard, or not very hard); physical activity (not meeting or meeting guidelines^20^); alcohol use in past year (yes or no); tobacco use (former, current, or never smoker); body mass index, which was calculated as weight in kilograms divided by height in meters squared (overweight or obese [≥25] or underweight or healthy weight [<25]); and self-rated health based on the 12-Item Short Form Health Survey^21^ (poor to fair or good to excellent).

Using a prior approach,^7^ the cumulative number of years with (1) high school diploma or GED certificate or less, (2) somewhat hard experience paying for basics, (3) not meeting physical activity guidelines, (4) alcohol use in past year or tobacco use, (5) overweight or obesity, and/or (6) fair or poor health was estimated using data collected at all examinations to account for potential changes in these covariates over time. For example, if a participant had healthy weight at baseline GXT and overweight at year 2 and each examination through year 20, the cumulative totals would be 2 years of healthy weight and 18 years of overweight. Factors that were hypothesized to exist on the causal pathway between cardiorespiratory fitness and the targeted health outcomes (eg, blood pressure or hypertension, fasting glucose level or diabetes, and cholesterol level or hyperlipidemia) were not considered as covariates.

### Statistical Analysis

Statistical analyses were conducted in October 2022 using R, version 4.1.3 (R Foundation for Statistical Computing). Initial analyses involved a descriptive content analysis that included univariate summaries of primary analysis variables at the baseline GXT and year 20 follow-up examination, overall and by race and sex subgroups. Continuous variables were summarized using means (SDs), and categorical variables were summarized using counts (percentages). Differences in these variables by race and sex were computed using Kruskal-Wallis rank sum test for continuous variables and Pearson χ^2^ tests for categorical variables. Two-sided *P* < .05 indicated statistical significance. The counts and percentages for cause of death, overall, and among race and sex subgroups were also summarized.

The count and proportion of missing values were identified overall, by GTX assessment, and by race and sex subgroups. Based on these data, we assumed the primary analysis variables were missing at random, and we conducted multiple imputation to obtain valid SEs for statistical inference. Multiple imputation with chained equations was applied, accounting for the longitudinal design of the study. Ten imputed data sets were formed by fitting a random forest to each variable with missing data separately, and then predictive mean matching was applied to generate imputed values.

The cumulative incidence of each outcome at 10 years (number of events [95% CI]) after the year 20 follow-up examination was estimated overall and by race and sex subgroups. Cox proportional hazards regression models were used to estimate hazard ratios (HRs) for each primary exposure with each outcome, adjusting for the variables. Models using percentage of early-adulthood cardiorespiratory fitness retained at year 20 were also adjusted for baseline GXT duration. In each model, a pooled Wald test comparing a model including natural cubic splines vs a simple linear term for the exposure was used to test whether the primary exposure had a nonlinear association with the outcome.^22,23^ Heterogeneity in the estimated association between each exposure and each outcome across race and sex subgroups was also examined.

Two sensitivity analyses were conducted. First, to examine the potential for reverse causation, participants who died or had a CVD event within the first 2 years of follow-up were removed. Second, a complete-case analysis was completed to determine the robustness of findings to imputation of missing cardiorespiratory fitness data.

## Results

The analytic sample included 4808 CARDIA study participants, of whom 2670 were females (56%) and 2138 were males (44%) with a mean (SD) age at baseline of 24.8 (3.7) years. Participants identified as either Black (2438 [51%]) or White (2370 [49%]), had a mean (SD) body mass index of 24.4 (4.9) at baseline GXT, completed the GXT protocol at least once (n = 10 860 tests), did not use β-blockers at the time of any GXT, achieved 85% or higher of their age-predicted maximal heart rate, and were alive and did not have CVD at the year 20 follow-up examination (Table 1 and Table 2).

**Table 1.** Inclusion Criteria for the Current Study

Criterion	No. of participants
CARDIA study participants enrolled at baseline GXT	5115
Did not withdraw consent	5114
Completed at least 1 GXT	5079
Not using β-blockers during GXT	5062
Alive and CVD-free at year 20 follow-up examination	4808

Abbreviations: CARDIA, Coronary Artery Risk Development in Young Adults; CVD, cardiovascular disease; GXT, graded exercise test.

**Table 2.** Participant Characteristics by Sex and Race at the Baseline GXT^a^^,^^b^^,^^c^

Characteristic	No. (%)				
	Overall	Black		White	
		Female	Male	Female	Male
No. of participants	4784	1389 (29)	1041 (22)	1268 (27)	1086 (23)
Field center clinic					
Birmingham, Alabama	1076 (22)	331 (24)	257 (25)	245 (19)	243 (22)
Chicago, Illinois	1048 (22)	303 (22)	214 (21)	274 (22)	257 (24)
Minneapolis, Minnesota	1318 (28)	296 (21)	274 (26)	398 (31)	350 (32)
Oakland, California	1342 (28)	459 (33)	296 (28)	351 (28)	236 (22)
Age, mean (SD), y	24.8 (3.7)	24.3 (3.8)	24.0 (3.7)	25.4 (3.4)	25.3 (3.4)
GXT duration, mean (SD), min	9.7 (3.1)	7.1 (2.1)	11.1 (2.6)	9.2 (2.4)	12.2 (2.5)
Resting					
Heart rate, bpm	78 (15)	82 (13)	70 (14)	83 (14)	75 (14)
Blood pressure, mm Hg					
Systolic	116 (13)	113 (11)	122 (13)	110 (10)	121 (12)
Diastolic	76 (11)	74 (10)	80 (12)	72 (9)	78 (11)
Heart rate at maximal exercise, bpm	179 (16)	174 (16)	177 (17)	181 (13)	185 (14)
Blood pressure at maximal exercise, mm Hg					
Systolic	179 (26)	165 (22)	197 (24)	166 (19)	196 (21)
Diastolic	78 (13)	77 (13)	81 (12)	74 (13)	79 (14)
Rating of perceived exertion at maximal exercise, mean (SD), points	17.9 (2.1)	17.4 (2.1)	17.7 (2.2)	18.2 (2.3)	18.5 (1.6)
Recovery heart rate: 2-min postexercise, bpm	136 (19)	132 (18)	131 (20)	139 (17)	142 (17)
Educational level					
≥Associate's degree	1565 (33)	274 (20)	188 (18)	605 (48)	498 (46)
≤High school diploma or GED certificate	3203 (67)	1112 (80)	849 (82)	661 (52)	581 (54)
Difficulty paying for basics					
Not very hard	3138 (66)	853 (61)	629 (61)	857 (68)	799 (74)
Somewhat hard	1639 (34)	534 (39)	410 (39)	408 (32)	287 (26)
Marital status					
Married or cohabitating	1058 (22)	271 (20)	189 (18)	355 (28)	243 (22)
Other^d^	3723 (78)	1117 (80)	851 (82)	912 (72)	843 (78)
BMI, mean (SD)	24.4 (4.9)	25.7 (6.4)	24.5 (4.2)	23.1 (4.3)	24.3 (3.5)
Physical activity					
Meeting guidelines	2811 (59)	519 (37)	756 (73)	738 (58)	798 (73)
Not meeting guidelines	1972 (41)	869 (63)	285 (27)	530 (42)	288 (27)
Self-reported health					
Excellent or good	4279 (90)	1160 (84)	914 (89)	1187 (94)	1018 (94)
Fair or poor	473 (10)	214 (16)	118 (11)	76 (6)	65 (6)
Alcohol use in past year					
No	653 (14)	297 (21)	151 (15)	119 (9)	86 (8)
Yes	4116 (86)	1091 (79)	887 (85)	1147 (91)	991 (92)
Tobacco use					
Current	1407 (30)	431 (31)	367 (36)	336 (27)	273 (25)
Former	631 (13)	116 (8)	94 (9)	253 (20)	168 (16)
Never	2714 (57)	837 (60)	569 (55)	673 (53)	635 (59)

Abbreviations: BMI, body mass index (calculated as weight in kilograms divided by height in meters squared); bpm, beats per minute; GED, General Educational Development; GXT, graded exercise test.

^a^ Data were from the baseline GXT (1985-1986) unless otherwise noted.

^b^ Baseline data are not presented for 24 participants (.05% of analytic sample) who did not complete the GXT at baseline but did complete the year 7 and/or year 20 follow-up examinations.

^c^ Calculated with Kruskal-Wallis rank sum test for continuous variables and Pearson χ^2^ test for categorical variables. *P* < .001 for all variables.

^d^ Other marital status included widowed, divorced, separated, and never married.

The number and percentage of completed GXTs by period (baseline, year 7, and year 20) and comparison of baseline characteristics by GXT completion status are shown in eTables 1 and 2 in Supplement 1. When comparing time-varying characteristics at baseline GXT (Table 2) vs year 20 follow-up examination (eTable 3 in Supplement 1) by race, Black males consistently had the highest proportions of lower educational level (82% at baseline and 56% at year 20) and current tobacco use (36% at baseline and 29% at year 20). Black females consistently had the highest proportions of not meeting physical activity guidelines (63% at baseline and 71% at year 20) and fair or poor self-rated health (16% at baseline and 17% at year 20) but had the lowest proportion of alcohol use in the past year (79% at baseline and 70% at year 20). Statistically significant differences in all characteristics, at both baseline GXT and year 20 follow-up examination, were noted by race or sex groups (Table 2).

During the 68 751 person-years of follow-up, 302 participants (6.3%) died and 274 participants (5.7%) had a CVD event after the year 20 follow-up examination. The leading causes of death were cancer and CVD (84 [28%] and 55 [18%], respectively) (eTable 4 in Supplement 1). The associations of early-adulthood cardiorespiratory fitness and the percentage of cardiorespiratory fitness retained in midlife with all-cause mortality and nonfatal or fatal CVD outcomes were linear, with no evidence of nonlinear effects (eFigure 1 in Supplement 1).

**Figure 1.**
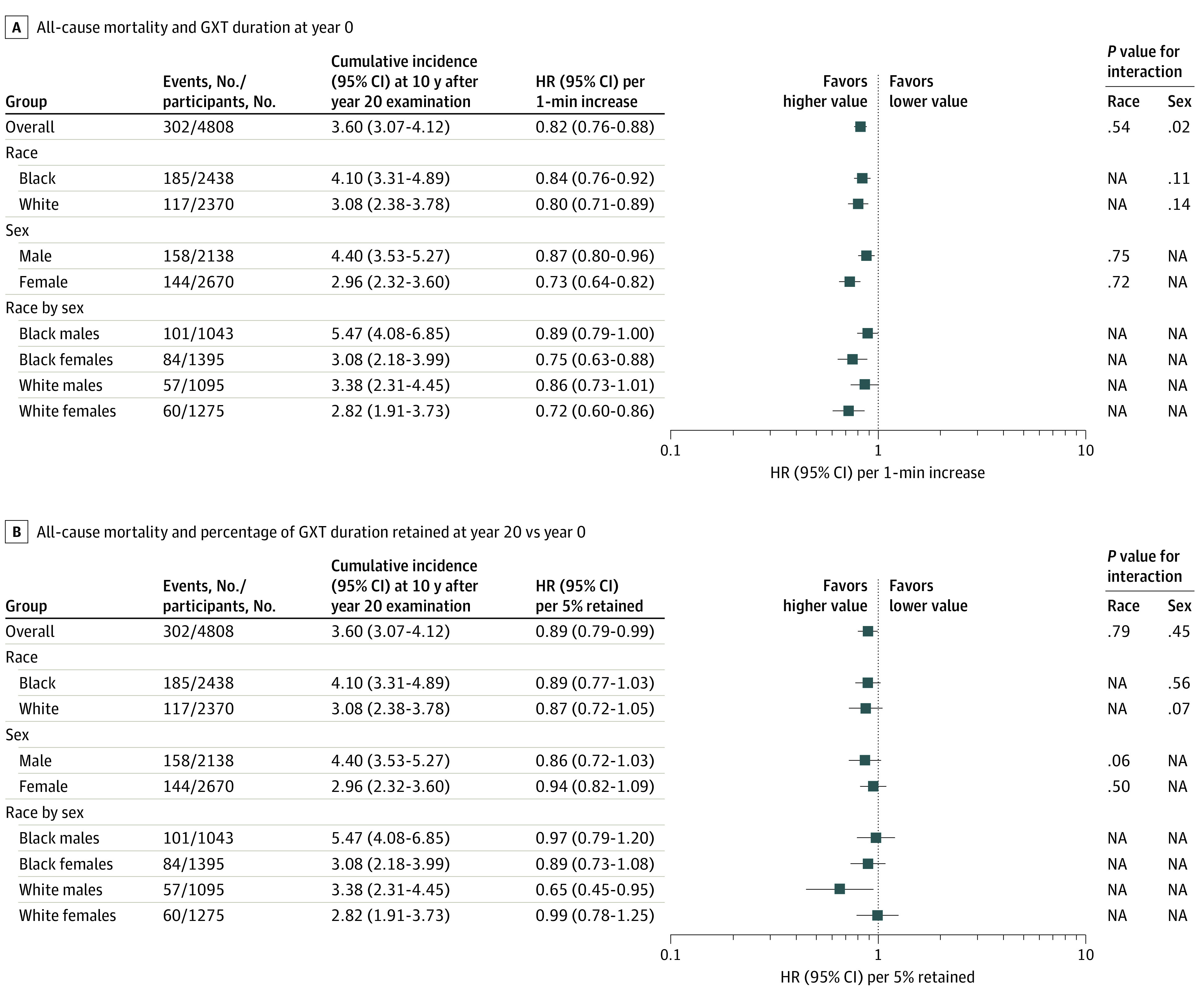
Multivariable-Adjusted Hazard Ratios (HRs) for All-Cause Mortality GXT indicates graded exercise test; NA, not applicable.

As shown in Figure 1, every 1-minute increment in baseline cardiorespiratory fitness was associated with a 19% lower risk of all-cause mortality (HR, 0.82; 95% CI, 0.76-0.88), with a significant interaction by sex but not by race. In females, each 1-minute increment in baseline cardiorespiratory fitness was associated with a 27% lower risk of all-cause mortality (HR, 0.73; 95% CI, 0.64-0.82); the association was attenuated in males but remained statistically significant (HR, 0.87; 95% CI, 0.80-0.96). Additionally, every 5% increment in early-adulthood cardiorespiratory fitness retained through year 20 was associated with an 11% lower risk of death (HR, 0.89; 95% CI, 0.79-0.99), with no evidence of effect modification by race or sex.

**Figure 2.**
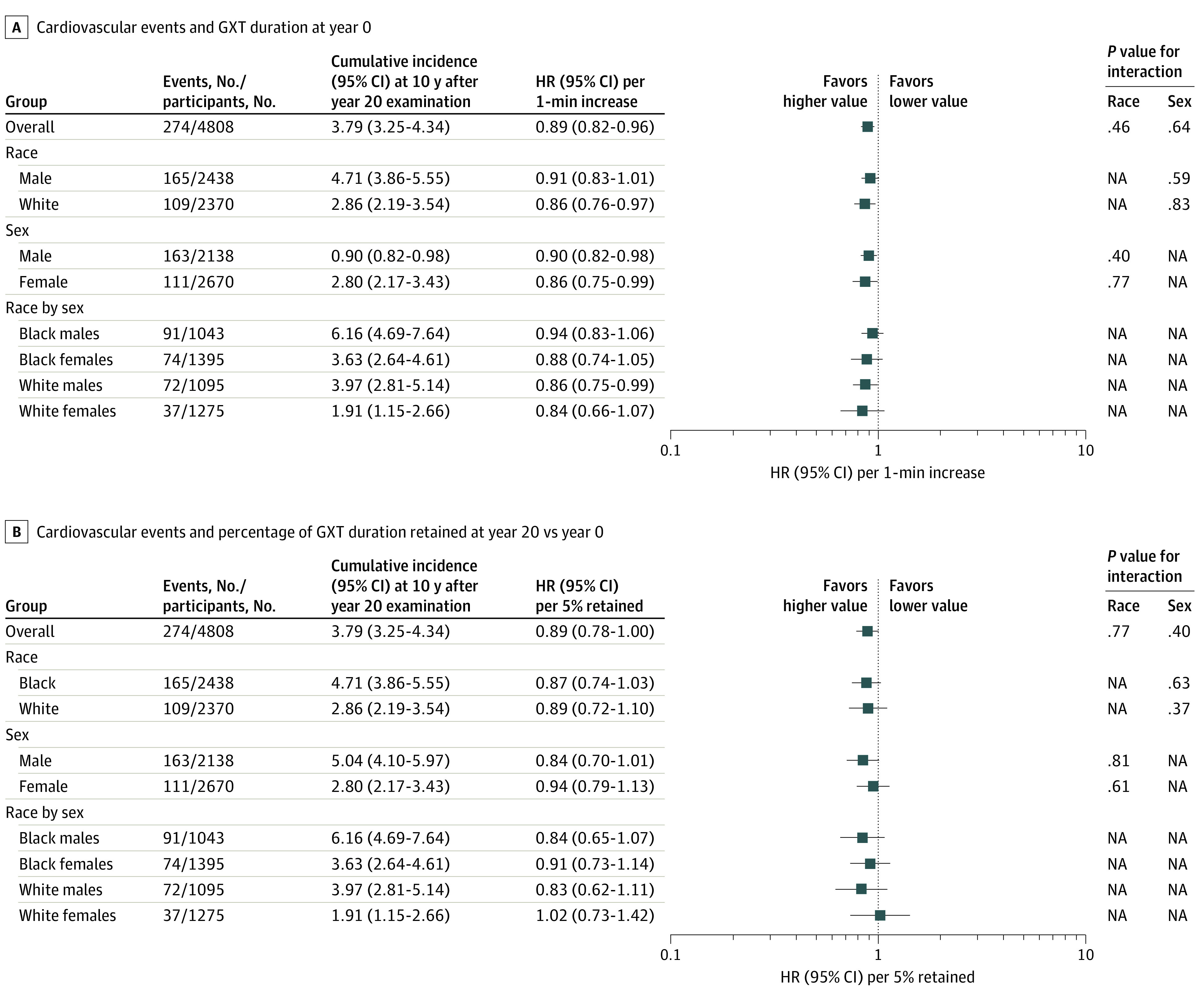
Multivariable-Adjusted Hazard Ratios (HRs) for Cardiovascular Events GXT indicates graded exercise test; NA, not applicable.

Figure 2 shows the associations of early-adulthood cardiorespiratory fitness and retention of cardiorespiratory fitness through year 20 with CVD events. Overall, every 1-minute increment in early-adulthood cardiorespiratory fitness was associated with a 11% lower risk of nonfatal or fatal CVD events (HR, 0.89; 95% CI, 0.82-0.96). There was no evidence of heterogeneity across race and sex subgroups in these associations. Also, the HR for nonfatal or fatal CVD event was 0.89 (95% CI, 0.78-1.00) per 5% increment in early-adulthood cardiorespiratory fitness retained in midlife. Similarly, there was no evidence of heterogeneity in these associations by race or sex.

In sensitivity analyses, findings were robust to the exclusion of participants who died or had a CVD event within the first 2 years of follow-up (eFigures 2 and 3 in Supplement 1). For example, early-adulthood fitness was associated with a reduced risk of all-cause mortality of 26% in females and 11% in males. Additionally, the findings were robust to the imputation of missing values according to a sensitivity analysis that was restricted to 2210 participants with complete data for age, sex, race, educational level, and cardiorespiratory fitness tests at baseline GXT and at year 7 and year 20 follow-up examinations (eTable 5 in Supplement 1). For example, early-adulthood fitness was associated with a 22% lower risk of all-cause mortality.

## Discussion

Findings from the CARDIA study complement the evidence demonstrating the inverse association of cardiorespiratory fitness with risk of all-cause mortality^2^ and nonfatal or fatal CVD events.^3^ The 3 principal findings of the present study were (1) higher early-adulthood cardiorespiratory fitness was associated with a reduced risk of premature death, an association that differed in magnitude by sex; (2) higher early-adulthood cardiorespiratory fitness was associated with a reduced risk of fatal or nonfatal CVD events; and (3) early-adulthood cardiorespiratory fitness retained through midlife was associated with a lower risk of all-cause mortality. Together, these findings provide novel evidence that the early-adulthood-to-midlife transition is a critical window for optimizing cardiorespiratory fitness to reduce risk of premature death and nonfatal or fatal CVD.

Prior studies^2,4,24^ have demonstrated significant associations of cardiorespiratory fitness levels with all-cause mortality; however, these studies were conducted in midlife or older adult populations. Specifically, a recent meta-analysis^2^ found that individuals in the highest tertile of baseline cardiorespiratory fitness had a 45% lower risk of all-cause mortality (relative risk, 0.55; 95% CI, 0.50-0.61) compared with individuals in the lowest tertile. Furthermore, each 1-MET higher cardiorespiratory fitness was associated with an 11% lower risk of all-cause mortality (relative risk, 0.89; 95% CI, 0.86-0.92).^2^ While these studies highlighted the importance of the starting point of cardiorespiratory fitness, the weighted mean age was 48.3 years across only 3 studies, including younger participants (aged ≤40 years).^4,24^ Studies^3,25^ have also found an inverse association between cardiorespiratory fitness measured at a single time point and CVD events, but again, these studies included older samples. Relying solely on evidence established in midlife and older adult populations can lead to spurious conclusions that place a higher emphasis on cardiorespiratory fitness during later life stages simply because cardiorespiratory fitness during earlier periods of life was unknown. In addition to the starting point, the current study also showed the importance of how much early-adulthood cardiorespiratory fitness was retained through midlife in association with subsequent risk of premature death.

This study augmented a prior CARDIA study analysis^4^ and included an additional measure of cardiorespiratory fitness that was collected during midlife and 9 additional years of follow-up. With this update, there was an association between early-adulthood cardiorespiratory fitness and all-cause mortality that was greater in magnitude than previously observed. Such an association may be due to a greater number of deaths that were unrelated to cardiorespiratory fitness (eg, AIDS [28%], homicide [16%], and suicide [7%]) experienced early in the CARDIA study.^26^ Since then, common causes of death have transitioned to those hypothesized to be physiologically related to cardiorespiratory fitness, including cancer and CVD. There was also a difference in the magnitude of the association between cardiorespiratory fitness and mortality by sex, with females having a larger risk reduction from higher cardiorespiratory fitness than males. Cardiorespiratory fitness levels in women have been estimated to be 25% lower than in men^27^; therefore, a 1-minute increment represented a higher proportion of total GXT duration, an estimate of maximal cardiorespiratory fitness. Additionally, since the year 20 follow-up examination, the most frequent cause of death was cancer (28%), with females experiencing a higher burden than males. Evidence supports the inverse association of physical activity, arguably the most important factor in cardiorespiratory fitness,^27^ with cancers that are most common in females.^28^

For CVD events, the findings also support the importance of the starting point of cardiorespiratory fitness. Given the physiological benefits of cardiorespiratory fitness, including (1) improved endothelial function, heart rate variability, and insulin sensitivity; (2) increased lean mass and mitochondrial and capillary density; and (3) reduced blood pressure, systemic inflammation, blood and plasma viscosity, and visceral adiposity,^29^ strategies to attenuate cardiorespiratory fitness reductions across the life course are also important. Specifically, enhanced cardiorespiratory fitness may be associated with a reduced risk or delayed onset of the accumulation of 1 or more traditional risk factors (eg, hypertension, hypercholesterolemia, and diabetes) that translate to a higher lifetime risk of CVD.^25,30^

### Limitations

These study findings should be interpreted within the context of several limitations. First, conclusions regarding a causal association of early-adulthood cardiorespiratory fitness and retention of early-adulthood cardiorespiratory fitness through midlife with mortality or CVD events cannot be established given that the CARDIA study is an observational study. Second, the cardiorespiratory fitness exposures were estimated based on test duration and did not include the collection of expired gases to obtain measured peak oxygen consumption, which is considered the gold standard measure. However, using treadmill duration and applying a threshold of at least 85% age-predicted maximal heart rate to infer maximal effort are standard approaches to estimating maximal cardiorespiratory fitness^5,31^ but may have resulted in the exclusion of tests among participants with chronotropic incompetence at the time of assessment. Third, while the low prevalence of outcomes (302 deaths [6.3% of sample] and 274 CVD events [5.7% of sample]) since the year 20 follow-up examination was expected given the age of the cohort, this prevalence may have limited our ability to detect heterogeneity in these associations by sex, race, and race or sex subgroups. Fourth, while the potential for reverse causation remains, participants were screened for medical eligibility prior to the GXT using American College of Sports Medicine criteria,^32^ and participants were excluded if they met contraindications that were predominantly cardiometabolic conditions. Fifth, the CARDIA study cohort includes participants who identified as being of Black or White race, which limits the generalizability of the findings to other racial and ethnic groups.

## Conclusions

Results from this cohort study complemented prior research supporting an inverse association of cardiorespiratory fitness with all-cause mortality and nonfatal or fatal CVD events. However, these associations were observed during an earlier period of the adult life course. Additional research is needed to clarify the association of cardiorespiratory fitness timing across the life course with risk of clinical outcomes.

## Notes

Supplement 1. **eTable 1.** Number and Percent of Completed GXT Tests by Exam Period, Overall and in Subgroups Defined by Race and Sex **eTable 2.** Participant Characteristics by GXT Disposition (Observed Versus Missing) at the Baseline (Year 0) Exam (1985-86) **eTable 3.** Participant Characteristics of the Entire Analytic Sample, and by Race/Sex Groups, at the Year 20 Follow-Up Exam (2005-06) **eTable 4.** Count (%) of Adjudicated Deaths Among CARDIA Participants Since the Year 20 Follow-Up Exam (2005-06) **eTable 5.** Multivariable-Adjusted Hazard Ratios for All-Cause Mortality and CVD Events Among Those With Complete Data (n=2,210) **eFigure 1.** Multivariable-Adjusted Hazard Ratios for All-Cause Mortality and Cardiovascular Events Using Natural Cubic Splines **eFigure 2.** Multivariable-Adjusted Hazard Ratios for All-Cause Mortality Excluding Individuals Who Died or Had a CVD Event in the First Two Years **eFigure 3.** Multivariable-Adjusted Hazard Ratios for Cardiovascular Events Excluding Individuals Who Died or Had a CVD Event in the First Two Years

Supplement 2.
Data Sharing Statement
