# Supplementary material for: Analysis of Cardiorespiratory Fitness in Early Adulthood and Midlife With All-Cause Mortality and Fatal or Nonfatal Cardiovascular Disease
Source: JAMA Netw Open. 2023 Feb 28;6(2):e230842. doi: 10.1001/jamanetworkopen.2023.0842 (PMC9975906; doi:10.1001/jamanetworkopen.2023.0842)
Supplement: Supplement 1. — eTable 1. Number and Percent of Completed GXT Tests by Exam Period, Overall and in Subgroups Defined by Race and Sex eTable 2. Participant Characteristics by GXT Disposition (Observed Versus Missing) at the Baseline (Year 0) Exam (1985-86) eTable 3. Participant Characteristics of the Entire Analytic Sample, and by Race/Sex Groups, at the Year 20 Follow-Up Exam (2005-06) eTable 4. Count (%) of Adjudicated Deaths Among CARDIA Participants Since the Year 20 Follow-Up Exam (2005-06) eTable 5. Multivariable-Adjusted Hazard Ratios for All-Cause Mortality and CVD Events Among Those With Complete Data (n=2,210) eFigure 1. Multivariable-Adjusted Hazard Ratios for All-Cause Mortality and Cardiovascular Events Using Natural Cubic Splines eFigure 2. Multivariable-Adjusted Hazard Ratios for All-Cause Mortality Excluding Individuals Who Died or Had a CVD Event in the First Two Years eFigure 3. Multivariable-Adjusted Hazard Ratios for Cardiovascular Events Excluding Individuals Who Died or Had a CVD Event in the First Two Years [file jamanetwopen-e230842-s001.pdf]

Supplemental Online Content

Pettee Gabriel K, Jaeger BC, Lewis CE, et al. Association of cardiorespiratory fitness in early adulthood and midlife with all-cause mortality and fatal or nonfatal cardiovascular disease. *JAMA Netw Open*. 2023;6(2):e230842. doi:10.1001/jamanetworkopen.2023.0842

eTable 1. Number and Percent of Completed GXT Tests by Exam Period, Overall and in Subgroups Defined by Race and Sex

eTable 2. Participant Characteristics by GXT Disposition (Observed Versus Missing) at the Baseline (Year 0) Exam (1985-86)

eTable 3. Participant Characteristics of the Entire Analytic Sample, and by Race/Sex Groups, at the Year 20 Follow-Up Exam (2005-06)

eTable 4. Count (%) of Adjudicated Deaths Among CARDIA Participants Since the Year 20 Follow-Up Exam (2005-06)

eTable 5. Multivariable-Adjusted Hazard Ratios for All-Cause Mortality and CVD Events Among Those With Complete Data (n=2,210)

eFigure 1. Multivariable-Adjusted Hazard Ratios for All-Cause Mortality and Cardiovascular Events Using Natural Cubic Splines

eFigure 2. Multivariable-Adjusted Hazard Ratios for All-Cause Mortality Excluding Individuals Who Died or Had a CVD Event in the First Two Years

eFigure 3. Multivariable-Adjusted Hazard Ratios for Cardiovascular Events Excluding Individuals Who Died or Had a CVD Event in the First Two Years

This supplementary material has been provided by the authors to give readers additional information about their work.

© 2023 Pettee Gabriel K et al. *JAMA Network Open.*

eTable 1. Number and Percent of Completed GXT Tests by Exam Period, Overall and in Subgroups Defined by Race and Sex

Group	Year 0	Year 7	Year 20
Overall	4,753 (99%)	3,397 (71%)	2,710 (59%)
Sex			
Male	2,115 (99%)	1,560 (73%)	1,177 (57%)
Female	2,638 (99%)	1,837 (69%)	1,533 (60%)
Race			
White	2,337 (99%)	1,836 (78%)	1,527 (66%)
Black	2,416 (99%)	1,561 (64%)	1,183 (51%)
Race-by-sex			
White Males	1,079 (99%)	903 (83%)	704 (66%)
Black Males	1,036 (100%)	657 (63%)	473 (47%)
Black Females	1,380 (99%)	904 (65%)	710 (54%)
White Females	1,258 (99%)	933 (74%)	823 (66%)

© 2023 Pettee Gabriel K et al. *JAMA Network Open.*

eTable 2. Participant Characteristics[1] by GXT Disposition (Observed Versus Missing[2]) at the Baseline (Year 0) Exam (1985-86)

Characteristic	Overall	GXT observed	GXT missing	p-value[1]
No. of participants	4,784	4,753	31	
Incident all-cause mortality[3] n (%)	173 (3.6)	171 (3.6)	2 (6.5)	0.3
Incident fatal / non-fatal cardiovascular event[3] n (%)				0.14
Censored	125 (2.6)	123 (2.6)	2 (6.5)	
No	4,479 (94)	4,452 (94)	27 (87)	
Yes	180 (3.8)	178 (3.7)	2 (6.5)	
Testing center, n (%)				0.008
Birmingham	1,076 (22)	1,076 (23)	0 (0)	
Chicago	1,048 (22)	1,042 (22)	6 (19)	
Minnesota	1,318 (28)	1,308 (28)	10 (32)	
Oakland	1,342 (28)	1,327 (28)	15 (48)	
Age, years	24.8 (3.7)	24.8 (3.7)	26.2 (3.1)	0.030
Education, n (%)				0.14
Associate's or more	1,565 (33)	1,551 (33)	14 (45)	
Highschool/GED or less	3,203 (67)	3,186 (67)	17 (55)	
Difficulty paying for basics, %				0.6
Not very hard	3,138 (66)	3,119 (66)	19 (61)	
Somewhat hard	1,639 (34)	1,627 (34)	12 (39)	
Marital status, n (%)				0.6
Married/Cohabitating	1,058 (22)	1,050 (22)	8 (26)	
Other	3,723 (78)	3,700 (78)	23 (74)	
Body mass index, kg/m^2	24.4 (4.9)	24.4 (4.9)	21.9 (3.4)	0.002
Physical Activity, n (%)				0.5
Meeting guidelines	2,811 (59)	2,791 (59)	20 (65)	
Not meeting guidelines	1,972 (41)	1,961 (41)	11 (35)	
Self-reported health, n (%)				0.5
Excellent or good	4,279 (90)	4,252 (90)	27 (87)	
Fair or poor	473 (10)	469 (9.9)	4 (13)	
Alcohol use (past year), n (%)				0.3
No	653 (14)	651 (14)	2 (6.5)	
Yes	4,116 (86)	4,087 (86)	29 (94)	
Smoking status, n (%)				0.7
Current	1,407 (30)	1,396 (30)	11 (35)	
Former	631 (13)	627 (13)	4 (13)	
Never smoked	2,714 (57)	2,698 (57)	16 (52)	

[1] Presented as percentages or mean (standard deviation).
[2] Wilcoxon rank sum test, Fisher's exact test, or Pearson's Chi-squared test.
[3] New cases accumulated after the Year 20 follow-up exam through August 2020.

© 2023 Pettee Gabriel K et al. *JAMA Network Open.*

eTable 3. Participant Characteristics[1] of the Entire Analytic Sample[2,3], and by Race/Sex Groups[4], at the Year 20 Follow-Up Exam (2005-06)

Characteristic	Overall	Black Women	Black Men	White Women	White Men	p-value[3]
No. of participants	4,632	1,323	1,006	1,238	1,065	
Testing center, n (%)						<0.001
Birmingham	1,030 (22)	308 (23)	249 (25)	238 (19)	235 (22)	
Chicago	1,027 (22)	293 (22)	209 (21)	269 (22)	256 (24)	
Minnesota	1,289 (28)	288 (22)	264 (26)	392 (32)	345 (32)	
Oakland	1,286 (28)	434 (33)	284 (28)	339 (27)	229 (22)	
Age, years	44.8 (3.7)	44.3 (3.8)	44.0 (3.7)	45.5 (3.4)	45.4 (3.4)	<0.001
GXT duration, minutes	7.2 (2.7)	5.0 (1.9)	7.7 (2.2)	7.1 (2.4)	9.2 (2.3)	<0.001
Resting Heart Rate, bpm	77 (17)	80 (26)	76 (13)	78 (12)	75 (12)	<0.001
Resting Systolic Blood Pressure, mmHg	126 (16)	125 (17)	132 (15)	119 (15)	130 (14)	<0.001
Resting Diastolic Blood Pressure, mmHg	79 (11)	80 (12)	82 (12)	77 (11)	80 (11)	<0.001
Heart Rate at Maximal Exercise, bpm	170 (15)	166 (16)	168 (18)	170 (14)	174 (13)	<0.001
Systolic Blood Pressure at Maximal Exercise, mmHg	178 (28)	168 (28)	194 (24)	164 (25)	190 (22)	<0.001
Diastolic Blood Pressure at Maximal Exercise, mmHg	78 (13)	79 (15)	83 (13)	75 (12)	79 (11)	<0.001
Rating of Perceived Exertion at Maximal Exercise	15.9 (2.3)	14.8 (2.7)	15.8 (2.3)	16.3 (2.0)	16.7 (1.7)	<0.001
Recovery Heart Rate (2-minutes post-exercise), bpm	130 (16)	128 (16)	127 (18)	129 (16)	134 (15)	<0.001
Education at year 20, n (%)						<0.001
≥ Associate's Degree	2,088 (63)	476 (53)	258 (44)	741 (77)	613 (73)	
≤ High school/GED	1,201 (37)	424 (47)	333 (56)	222 (23)	222 (27)	
Difficulty paying for basics, n (%)						<0.001
Not very hard	2,490 (76)	578 (64)	425 (72)	780 (81)	707 (85)	
Somewhat hard	796 (24)	321 (36)	166 (28)	182 (19)	127 (15)	
Marital status, n (%)						<0.001
Married/Cohabitating	1,856 (56)	361 (40)	295 (50)	641 (67)	559 (67)	
Other	1,432 (44)	537 (60)	297 (50)	322 (33)	276 (33)	
Body mass index, kg/m^2	29 (7)	32 (8)	29 (6)	27 (7)	28 (5)	<0.001
Physical activity, n (%)						<0.001
Meeting guidelines	1,560 (48)	259 (29)	328 (55)	465 (48)	508 (61)	
Not meeting guidelines	1,723 (52)	638 (71)	264 (45)	495 (52)	326 (39)	
Self-reported health, n (%)						<0.001

© 2023 Pettee Gabriel K et al. *JAMA Network Open.*

Characteristic	Overall	Black Women	Black Men	White Women	White Men	p-value[3]
Excellent or good	2,915 (90)	727 (83)	513 (87)	899 (94)	776 (94)	
Fair or poor	339 (10)	152 (17)	78 (13)	57 (6.0)	52 (6.3)	
Alcohol use (past year), n (%)						<0.001
No	666 (21)	268 (30)	151 (26)	135 (14)	112 (14)	
Yes	2,560 (79)	613 (70)	422 (74)	817 (86)	708 (86)	
Smoking status, n (%)						<0.001
Current	632 (19)	207 (23)	171 (29)	126 (13)	128 (16)	
Former	621 (19)	111 (12)	67 (11)	273 (28)	170 (21)	
Never smoked	2,018 (62)	578 (65)	354 (60)	559 (58)	527 (64)	

[1] Presented as percentages or mean (standard deviation)
[2] Data are from the Year 20 exam unless otherwise noted.
[3] Year 20 data are not represented for 174 participants (3.6% of analytic sample) who did not complete the graded exercise test at the Year 20 follow-up exam but did complete the protocol at Baseline (Year 0) and/or Year 7 follow-up exams.
[4] Kruskal-Walis rank sum test or Pearson's Chi-squared test

© 2023 Pettee Gabriel K et al. *JAMA Network Open.*

eTable 4. Count (%) of Adjudicated Deaths Among CARDIA Participants Since the Year 20 Follow-Up Exam (2005-06)

**Cause of death**	**Overall**	**Race**		**Sex**		**Race by Sex**			
	**Overall (N = 302)**	**Black (N = 185)**	**White (N = 117)**	**Female (N = 144)**	**Male (N = 158)**	**Black Female (N = 84)**	**Black Male (N = 101)**	**White Female (N = 60)**	**White Male (N = 57)**
AIDS	10 (3.3%)	7 (3.8%)	3 (2.6%)	0 (0%)	10 (6.3%)	0 (0%)	7 (6.9%)	0 (0%)	3 (5.3%)
Asthma	2 (0.66%)	2 (1.1%)	0 (0%)	1 (0.69%)	1 (0.63%)	1 (1.2%)	1 (0.99%)	0 (0%)	0 (0%)
Cancer	84 (28%)	47 (25%)	37 (32%)	53 (37%)	31 (20%)	29 (35%)	18 (18%)	24 (40%)	13 (23%)
Total CVD	55 (18.2%)	31 (16.8%)	24 (20.5%)	18 (12.5%)	37 (23.4%)	11 (13.1%)	20 (19.8%)	7 (11.7%)	17 (29.8%)
ASCVD, CHD	27 (8.9%)	10 (5.4%)	17 (15%)	8 (5.6%)	19 (12%)	4 (4.8%)	6 (5.9%)	4 (6.7%)	13 (23%)
ASCVD, other	3 (0.99%)	3 (1.6%)	0 (0%)	1 (0.69%)	2 (1.3%)	1 (1.2%)	2 (2.0%)	0 (0%)	0 (0%)
ASCVD, stroke	11 (3.6%)	7 (3.8%)	4 (3.4%)	6 (4.2%)	5 (3.2%)	4 (4.8%)	3 (3.0%)	2 (3.3%)	2 (3.5%)
CVD, non-atherosclerotic	14 (4.6%)	11 (5.9%)	3 (2.6%)	3 (2.1%)	11 (7.0%)	2 (2.4%)	9 (8.9%)	1 (1.7%)	2 (3.5%)
Pulmonary embolism	2 (0.66%)	1 (0.54%)	1 (0.85%)	2 (1.4%)	0 (0%)	1 (1.2%)	0 (0%)	1 (1.7%)	0 (0%)
Diabetes	1 (0.33%)	1 (0.54%)	0 (0%)	1 (0.69%)	0 (0%)	1 (1.2%)	0 (0%)	0 (0%)	0 (0%)
Kidney disease	7 (2.3%)	6 (3.2%)	1 (0.85%)	5 (3.5%)	2 (1.3%)	4 (4.8%)	2 (2.0%)	1 (1.7%)	0 (0%)
Liver disease	16 (5.3%)	6 (3.2%)	10 (8.5%)	8 (5.6%)	8 (5.1%)	3 (3.6%)	3 (3.0%)	5 (8.3%)	5 (8.8%)
Other	39 (13%)	28 (15%)	11 (9.4%)	17 (12%)	22 (14%)	11 (13%)	17 (17%)	6 (10%)	5 (8.8%)
Sepsis	8 (2.6%)	7 (3.8%)	1 (0.85%)	3 (2.1%)	5 (3.2%)	2 (2.4%)	5 (5.0%)	1 (1.7%)	0 (0%)
Homicide	8 (2.6%)	8 (4.3%)	0 (0%)	0 (0%)	8 (5.1%)	0 (0%)	8 (7.9%)	0 (0%)	0 (0%)
Suicide	12 (4.0%)	2 (1.1%)	10 (8.5%)	8 (5.6%)	4 (2.5%)	0 (0%)	2 (2.0%)	8 (13%)	2 (3.5%)
Unintentional injury	20 (6.6%)	12 (6.5%)	8 (6.8%)	7 (4.9%)	13 (8.2%)	4 (4.8%)	8 (7.9%)	3 (5.0%)	5 (8.8%)
Unknown	38 (13%)	27 (15%)	11 (9.4%)	21 (15%)	17 (11%)	17 (20%)	10 (9.9%)	4 (6.7%)	7 (12%)

© 2023 Pettee Gabriel K et al. *JAMA Network Open.*

eTable 5. Multivariable-Adjusted Hazard Ratios for All-Cause Mortality and CVD Events Among Those With Complete Data (n=2,210)[1]

	**All-cause Mortality**			**Cardiovascular Events**		
	N Events / participants	Cumulative Incidence[2] (95% CI)	Hazards Ratio (95% CI)	N Events / participants	Cumulative Incidence[2] (95% CI)	Hazards Ratio (95% CI)
GXT Duration at Y0 per 1-minute	90 / 2,210	2.40 (1,76, 3.04)	0.78 (0.67, 0.91)	111 / 2,210	2.97 (2.26, 3.68)	0.91 (0.79, 1.04)
% GXT Duration Retained at Y20 versus Y0, per 5% retained	90 / 2,210	2.40 (1,76, 3.04)	0.89 (0.73, 1.09)	111 / 2,210	2.97 (2.26, 3.68)	0.85 (0.70, 1.03)

[1] Complete-cases defined as those with available data for GXT duration, age, sex, race, education, and enrolling CARDIA clinic.
[2] At 10 years post Y20 exam.

© 2023 Pettee Gabriel K et al. *JAMA Network Open.*

eFigure 1. Multivariable-Adjusted Hazard Ratios for All-Cause Mortality and Cardiovascular Events Using Natural Cubic Splines

Grey shaded areas represent 95% confidence intervals for the point-estimate.

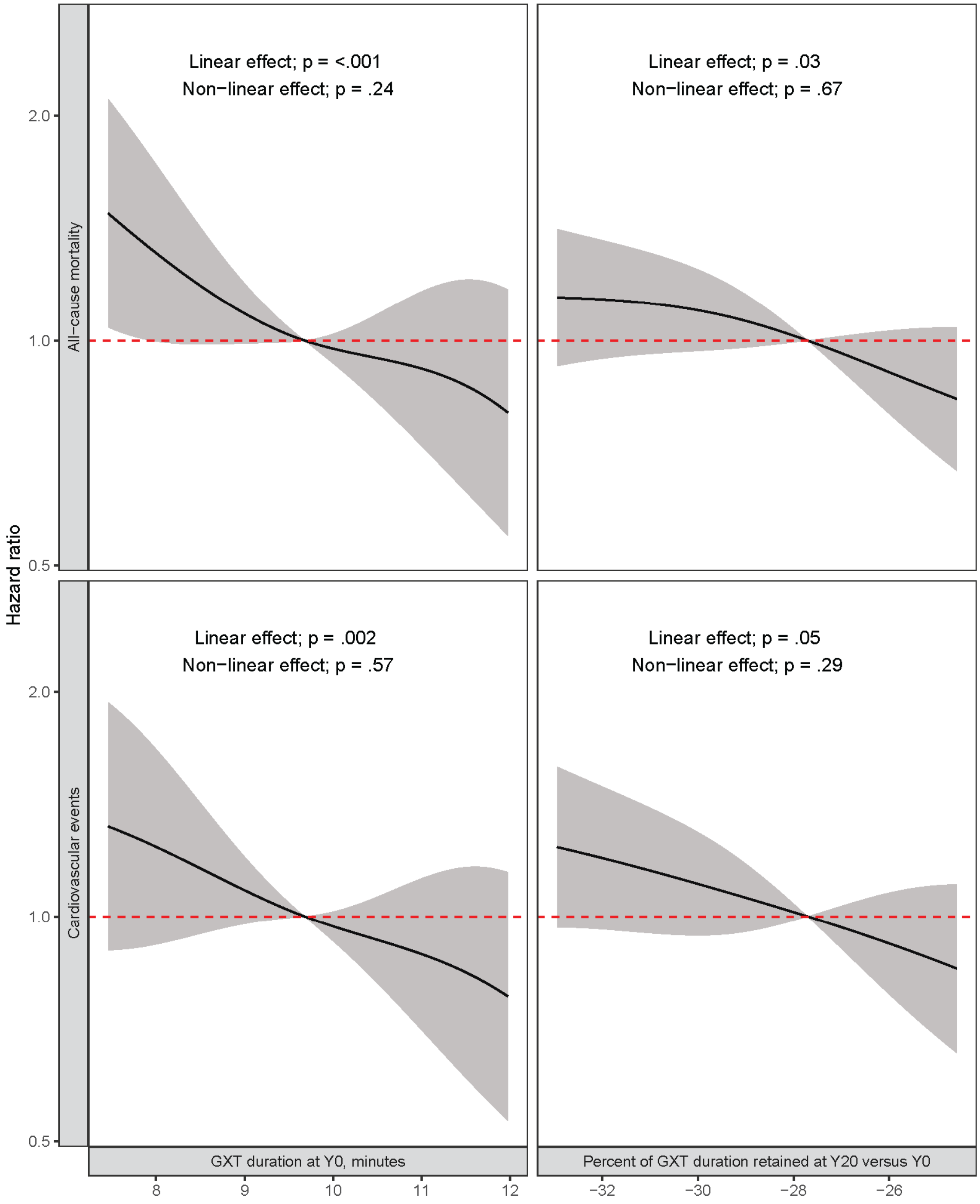

© 2023 Pettee Gabriel K et al. *JAMA Network Open.*

eFigure 2. Multivariable-Adjusted Hazard Ratios for All-Cause Mortality Excluding Individuals Who Died or Had a CVD Event in the First Two Years

All−cause mortality and GXT duration at Y0

Group	N events / participants	Cumulative Incidence (95% CI)<br>*At 10 years post Y20 exam*	Hazard ratio (95% CI)<br>*Per 1 minute increase*	P−value for interaction<br>*Race*	 *Sex*
Overall	261 / 4,748	2.80 (2.33, 3.27)	0.84 (0.77, 0.90)	.85	.03
Race					
Black	160 / 2,399	3.17 (2.47, 3.87)	0.85 (0.77, 0.93)	−−	.12
White	101 / 2,349	2.43 (1.80, 3.05)	0.83 (0.74, 0.94)	−−	.30
Sex					
Men	138 / 2,109	3.56 (2.77, 4.35)	0.89 (0.81, 0.98)	.78	−−
Women	123 / 2,639	2.20 (1.64, 2.76)	0.74 (0.65, 0.85)	.96	−−
Race by Sex					
Black Men	86 / 1,022	4.21 (2.98, 5.44)	0.90 (0.79, 1.03)	−−	−−
Black Women	74 / 1,377	2.40 (1.59, 3.20)	0.76 (0.64, 0.90)	−−	−−
White Men	52 / 1,087	2.94 (1.94, 3.95)	0.88 (0.74, 1.04)	−−	−−
White Women	49 / 1,262	1.98 (1.21, 2.75)	0.76 (0.62, 0.93)	−−	−−

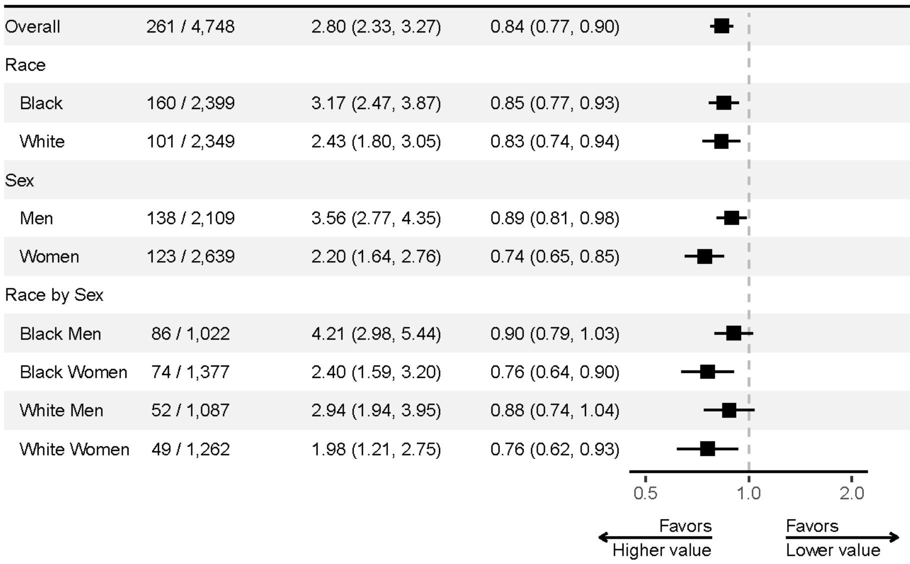

All−cause mortality and percent of GXT duration retained at Y20 versus Y0

Group	N events / participants	Cumulative Incidence (95% CI)<br>*At 10 years post Y20 exam*	Hazard ratio (95% CI)<br>*Per 5% retained*	P−value for interaction<br>*Race*	 *Sex*
Overall	261 / 4,748	2.80 (2.33, 3.27)	0.85 (0.75, 0.96)	.36	.84
Race					
Black	160 / 2,399	3.17 (2.47, 3.87)	0.88 (0.76, 1.03)	−−	.37
White	101 / 2,349	2.43 (1.80, 3.05)	0.78 (0.63, 0.96)	−−	.19
Sex					
Men	138 / 2,109	3.56 (2.77, 4.35)	0.86 (0.71, 1.05)	.046	−−
Women	123 / 2,639	2.20 (1.64, 2.76)	0.87 (0.74, 1.02)	.88	−−
Race by Sex					
Black Men	86 / 1,022	4.21 (2.98, 5.44)	1.00 (0.79, 1.26)	−−	−−
Black Women	74 / 1,377	2.40 (1.59, 3.20)	0.86 (0.70, 1.06)	−−	−−
White Men	52 / 1,087	2.94 (1.94, 3.95)	0.63 (0.42, 0.94)	−−	−−
White Women	49 / 1,262	1.98 (1.21, 2.75)	0.86 (0.65, 1.14)	−−	−−

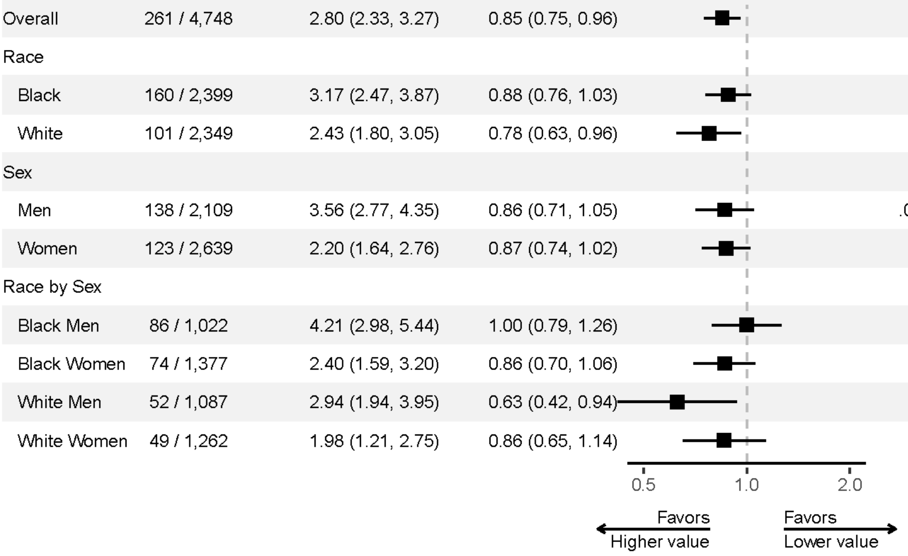

© 2023 Pettee Gabriel K et al. *JAMA Network Open.*

eFigure 3. Multivariable-Adjusted Hazard Ratios for Cardiovascular Events Excluding Individuals Who Died or Had a CVD Event in the First Two Years

Cardiovascular events and GXT duration at Y0

Group	N events / participants	Cumulative Incidence (95% CI)	Hazard ratio (95% CI)	P−value for interaction	
		At 10 years post Y20 exam	*Per 1 minute increase*	*Race*	*Sex*
Overall	241 / 4,748	3.13 (2.63, 3.63)	0.90 (0.83, 0.97)	.51	.89
Race					
Black	141 / 2,399	3.76 (2.99, 4.52)	0.92 (0.83, 1.03)	--	.79
White	100 / 2,349	2.49 (1.86, 3.12)	0.87 (0.77, 0.99)	--	.83
Sex					
Men	146 / 2,109	4.27 (3.40, 5.14)	0.90 (0.82, 0.99)	.45	--
Women	95 / 2,639	2.22 (1.65, 2.78)	0.90 (0.77, 1.05)	.88	--
Race by Sex					
Black Men	79 / 1,022	5.07 (3.71, 6.42)	0.94 (0.82, 1.07)	--	--
Black Women	62 / 1,377	2.79 (1.91, 3.66)	0.90 (0.74, 1.09)	--	--
White Men	67 / 1,087	3.53 (2.43, 4.64)	0.87 (0.75, 1.00)	--	--
White Women	33 / 1,262	1.59 (0.90, 2.29)	0.89 (0.69, 1.16)	--	--

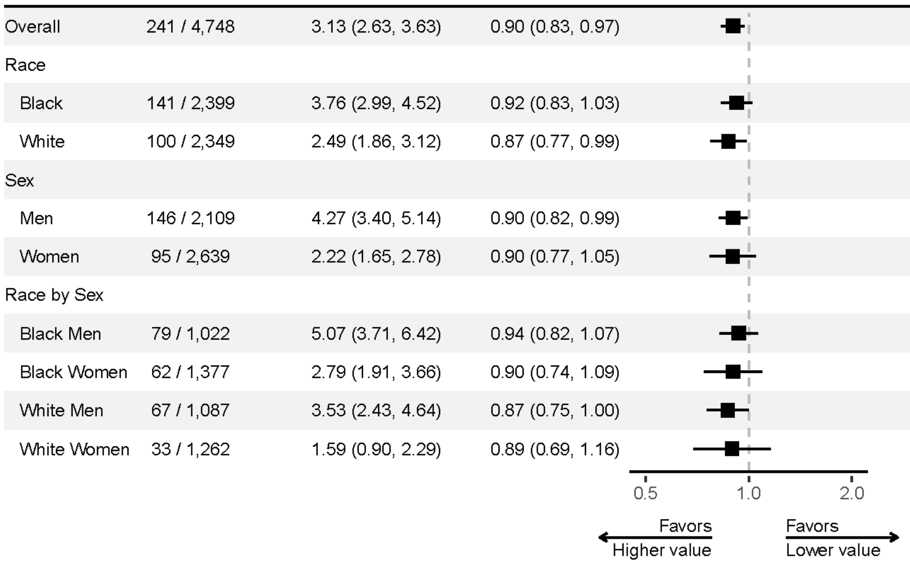

Cardiovascular events and percent of GXT duration retained at Y20 versus Y0

Group	N events / participants	Cumulative Incidence (95% CI)	Hazard ratio (95% CI)	P−value for interaction	
		At 10 years post Y20 exam	*Per 5% retained*	*Race*	*Sex*
Overall	241 / 4,748	3.13 (2.63, 3.63)	0.87 (0.76, 0.99)	.44	.29
Race					
Black	141 / 2,399	3.76 (2.99, 4.52)	0.90 (0.76, 1.07)	--	.44
White	100 / 2,349	2.49 (1.86, 3.12)	0.80 (0.64, 1.01)	--	.49
Sex					
Men	146 / 2,109	4.27 (3.40, 5.14)	0.81 (0.67, 0.98)	.67	--
Women	95 / 2,639	2.22 (1.65, 2.78)	0.94 (0.77, 1.15)	.76	--
Race by Sex					
Black Men	79 / 1,022	5.07 (3.71, 6.42)	0.83 (0.64, 1.09)	--	--
Black Women	62 / 1,377	2.79 (1.91, 3.66)	0.97 (0.76, 1.23)	--	--
White Men	67 / 1,087	3.53 (2.43, 4.64)	0.76 (0.56, 1.03)	--	--
White Women	33 / 1,262	1.59 (0.90, 2.29)	0.90 (0.62, 1.31)	--	--

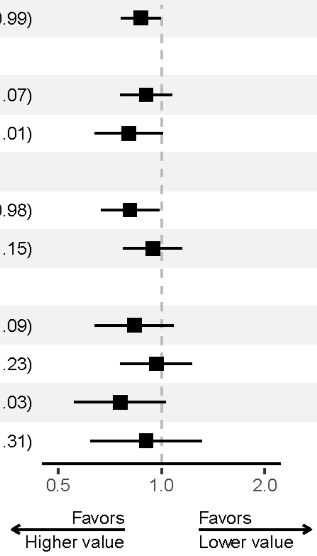

© 2023 Pettee Gabriel K et al. *JAMA Network Open.*
