# Supplementary material for: Analysis of Cardiorespiratory Fitness in Early Adulthood and Midlife With All-Cause Mortality and Fatal or Nonfatal Cardiovascular Disease
Source: JAMA Netw Open. 2023 Feb 28;6(2):e230842. doi: 10.1001/jamanetworkopen.2023.0842 (PMC9975906; doi:10.1001/jamanetworkopen.2023.0842)
Supplement: Supplement 2. — Data Sharing Statement [file jamanetwopen-e230842-s002.pdf]

Data Sharing Statement

Pettee Gabriel. Analysis of Cardiorespiratory Fitness in Early Adulthood and Midlife With All-Cause Mortality and Fatal or Nonfatal Cardiovascular Disease. *JAMA Netw Open*. Published February 28, 2023. doi:10.1001/jamanetworkopen.2023.0842

Data
Data available: Yes
Data types: Deidentified participant data
How to access data: CARDIA data are available upon reasonable request from the CARDIA Coordinating Center. CARDIA investigators are eager to collaborate with investigators interested in using CARDIA data. Please see the CARDIA website (https://www.cardia.dopm.uab.edu) for publications policies and for a list of CARDIA investigators. CARDIA data are also publicly available on the NIH-supported BioLINCC and dbGaP platforms.
When available: With publication

Supporting Documents
Document types: Statistical/analytic code
How to access documents: https://github.com/bcjaeger/CARDIA---GXT-and-CVD
When available: With publication

Additional Information
Who can access the data: researchers whose proposed use of the data has been approved
Types of analyses: for any specified purpose
Mechanisms of data availability: after approval of a proposal and signed data use agreement
